# The effect of high-dietary K^+^ (HK) on Kir4.1/Kir5.1 and ROMK in the distal convoluted tubule (DCT) is not affected by gender and Cl^−^ content of the diet

**DOI:** 10.3389/fphys.2022.1039029

**Published:** 2022-11-09

**Authors:** Xin-Xin Meng, Hao Zhang, Gui-Lin Meng, Shao-Peng Jiang, Xin-Peng Duan, Wen-Hui Wang, Ming-Xiao Wang

**Affiliations:** ^1^ Department of Physiology, Zhuhai Campus of Zunyi Medical University, Zhuhai, China; ^2^ Department of Pharmacology, New York Medical College, Valhalla, NY, United States

**Keywords:** renal K^+^ excretion, thiazide-sensitive, NCC, KCNJ1, KCNJ10, Kcnj16

## Abstract

Basolateral potassium channels in the distal convoluted tubule (DCT) are composed of inwardly-rectifying potassium channel 4.1 (Kir4.1) and Kir5.1. Kir4.1 interacts with Kir5.1 to form a 40 pS K^+^ channel which is the only type K^+^ channel expressed in the basolateral membrane of the DCT. Moreover, Kir4.1/Kir5.1 heterotetramer plays a key role in determining the expression and activity of thiazide-sensitive Na-Cl cotransport (NCC). In addition to Kir4.1/Kir5.1, Kir1.1 (ROMK) is expressed in the apical membrane of the late DCT (DCT2) and plays a key role in mediating epithelial Na^+^ channel (ENaC)-dependent K^+^ excretion. High dietary-K^+^-intake (HK) stimulates ROMK and inhibits Kir4.1/Kir5.1 in the DCT. Inhibition of Kir4.1/Kir5.1 is essential for HK-induced suppression of NCC whereas the stimulation of ROMK is important for increasing ENaC-dependent K^+^ excretion during HK. We have now used the patch-clamp-technique to examine whether gender and Cl^−^ content of K^+^-diet affect HK-induced inhibition of basolateral Kir4.1/Kir5.1 and HK-induced stimulation of ROMK. Single-channel-recording shows that basolateral 40 pS K^+^ channel (Kir4.1/Kir5.1) activity of the DCT defined by NP_o_ was 1.34 (1% KCl, normal K, NK), 0.95 (5% KCl) and 1.03 (5% K^+^-citrate) in male mice while it was 1.47, 1.02 and 1.05 in female mice. The whole-cell recording shows that Kir4.1/Kir5.1-mediated-K^+^ current of the early-DCT (DCT1) was 1,170 pA (NK), 725 pA (5% KCl) and 700 pA (5% K^+^-citrate) in male mice whereas it was 1,125 pA, 674 pA and 700 pA in female mice. Moreover, K^+^-currents (I_K_) reversal potential of DCT (an index of membrane potential) was -63 mV (NK), −49 mV (5% KCl) and −49 mV (5% K-citrate) in the male mice whereas it was -63 mV, −50 mV and −50 mV in female mice. Finally, TPNQ-sensitive whole-cell ROMK-currents in the DCT2 /initial-connecting tubule (CNT) were 910 pA (NK), 1,520 pA (5% KCl) and 1,540 pA (5% K^+^−citrate) in male mice whereas the ROMK-mediated K^+^ currents were 1,005 pA, 1,590 pA and 1,570 pA in female mice. We conclude that the effect of HK intake on Kir4.1/Kir5.1 of the DCT and ROMK of DCT2/CNT is similar between male and female mice. Also, Cl^−^ content in HK diets has no effect on HK-induced inhibition of Kir4.1/Kir5.1 of the DCT and HK-induced stimulation of ROMK in DCT2/CNT.

## Introduction

Previous studies have detected Kir4.1/Kir5.1 activity in the late thick ascending limb (TAL), DCT, CNT and cortical collecting duct (CCD) of the mammalian kidney (Lourdel et al., 2002; Lachheb et al., 2008; Zhang et al., 2015; Su et al., 2016; Su and Wang, 2016; Cuevas et al., 2017; Palygin et al., 2017; Wang et al., 2018a). These studies have also shown that Kir4.1/Kir5.1 activity is responsible for the basolateral K^+^ conductance in the DCT (Zhang et al., 2014; Cuevas et al., 2017; Wang et al., 2018a). Thus, Kir4.1/Kir5.1 heterotetramer plays a key role in determining the negativity of the membrane potential in the DCT under physiological conditions (Zhang et al., 2014; Su et al., 2019). In addition to Kir4.1/Kir5.1, ROMK activity/expression is detected in the apical membrane of late DCT (DCT2) although ROMK immunostaining is also detected in the intracellular space of DCT1 (Obermuller et al., 1995; Wade et al., 2011; Wu et al., 2018; Wu et al., 2020a). In addition to performing NCC-dependent Na^+^ absorption (Ellison et al., 1987), DCT is also the nephron segment participating in ENaC and ROMK dependent K^+^ excretion (Wade et al., 2011; Nesterov et al., 2012; Su et al., 2019; Wu et al., 2020a; Zhang et al., 2021). Long-term HK intake has been shown to inhibit Na^+^ absorption and stimulate K^+^ excretion in the DCT by inhibiting thiazide-sensitive NCC along whole DCT and stimulating ROMK and ENaC in the late DCT (DCT2), CNT and CCD (Hebert et al., 2005; Vallon et al., 2009; Sorensen et al., 2013; Rengarajan et al., 2014; Terker et al., 2015; Terker et al., 2016; Yang et al., 2018; Yang et al., 2021). We have demonstrated that increased dietary K^+^ intake inhibited Kir4.1/Kir5.1 of the DCT (Wang et al., 2018a). Moreover, the inhibition of Kir4.1/Kir5.1 of the DCT is essential for HK-induced inhibition of NCC. The inhibition of NCC should increase Na^+^ delivery to the aldosterone-sensitive distal nephron (ASDN) thereby enhancing ENaC-dependent K^+^ excretion through ROMK which is also stimulated by increased dietary K^+^ intake (Wang, 1995; Yang et al., 2021; Nesterov et al., 2022). Moreover, NCC inhibition-induced increase in volume delivery should also stimulate the flow-induced K^+^ excretion by Ca^2+^-dependent big-conductance K^+^ channel (Satlin, 2004). Thus, the inhibition of Kir4.1/Kir5.1 in the DCT and stimulation of ROMK and ENaC in the DCT2 and CNT work in concert to facilitate K^+^ excretion during increased dietary K^+^ intake.

Although the HK-induced inhibition of the basolateral Kir4.1/Kir5.1 activity of the DCT and HK-induced stimulation of ROMK are well established, it is not investigated whether there is a gender difference regarding the effect of HK on Kir4.1/Kir5.1 and ROMK because animals used in the previous studies are either male only or a mixture population (Hebert et al., 2005; Frindt et al., 2009; Wang et al., 2018a; Nesterov et al., 2022). Also, previous experiments in which the effect of HK intake on Kir4.1/Kir5.1 or ROMK was tested have often used the HK diets containing high Cl^−^ content (Palmer et al., 1994; Wei et al., 2001; Sterling et al., 2004; Frindt et al., 2009; Wang et al., 2018a; Xiao et al., 2021). Thus, it is not systemically tested whether Cl^−^ content of the HK diets could have an effect on HK-induced inhibition of Kir4.1/Kir5.1 of the DCT and on HK-induced stimulation of ROMK. Therefore, the aims of the present study are: 1) Test whether there is a gender difference for the effect of HK intake on Kir4.1/Kir5.1 and ROMK; 2) Assess whether the effect of HK on Kir4.1/Kir5.1 and ROMK is modified by Cl^−^ contents in the HK diet.

## Materials and methods

### Preparation of the distal convoluted tubule

We used 8–10-week-old C57/BL6 mice obtained from Zhuhai campus of Zunyi Medical University. The mice were kept on standard rodent chow containing 1% KCl, 5% KCl or 5% K^+^-citrate for 7 days. For dissecting renal DCT in male and female mice, we euthanized the mice with CO_2_ inhalation plus cervical dislocation followed by opening the abdomen of the mice to expose the left kidney. The left kidney was then perfused with 2 ml L-15 medium (Life Technology) containing Type 2 collagenase (250 unit/ml). After removing the collagenase-perfused kidney, we cut the renal cortex into small pieces which were further incubated in collagenase-containing L-15 media for 40–50 min at 37°C. We washed the tissue three times with fresh L-15 medium and transferred the renal tissue to an ice-cold chamber for dissection. We placed the isolated DCT on a small cover glass coated with poly-lysine and move the cover glass to a chamber mounted on an inverted microscope.

## Ethical approval

The protocol for animal use has been approved by independent Institutional Animal Use and Care Committee at NYMC and by Ethics Committee of Zunyi Medical University Zhuhai Campus.

### Manufacture of patch-clamp pipette

We used a Narishige electrode puller (Narishige, Japan) to make the patch-clamp pipettes from Borosilicate glass (1.7-mm OD). The pipette was filled with (in mM) 140 KCl, 1.8 MgCl_2_ and 10 HEPES (titrated with KOH to pH 7.4) and the resistance of the pipette was 5 MΩ (for single channel recording) or 2 MΩ (for whole-cell recording), respectively.

### Whole cell recording

Whole-cell TPNQ-sensitive K^+^ currents (ROMK) and Ba^2+^-sensitive Kir4.1/Kir5.1-mediated K^+^ currents were measured with an Axon 200B amplifier. The K^+^ currents were low-pass filtered at 1 KHz, and digitized by an Axon interface (Digidata 1550B) with sampling rate of 4 KHz. We measured the ROMK-mediated K^+^ currents with gap-free protocol in split-open DCT2 (last 100 μm DCT before the start of the CNT). We used morphological appearance to determine the end of DCT whose diameter was larger than the CNT. However, we could not exclude the possibility that some experiments were actually performed in the early CNT. Accordingly, we have stated that ROMK-mediated K^+^ currents were performed in the DCT2/CNT. We used.

Step-protocol or ramp-protocol to measure Kir4.1/Kir5.1 mediated K^+^ currents in early part of the DCT (DCT1) which was identified in the first 100 μm after glomerular attachment. After forming a high resistance seal, we monitored the membrane capacitance until the whole-cell patch configuration was formed. We filled the tip of the pipette with a solution containing (in mM) 140 KCl, 2 MgATP, 1 EGTA, and 10 HEPES (pH 7.4) and then back-filled the pipette with the pipette solution containing amphotericin B (20 μg/0.1 ml). The tubule was perfused in a bath solution contains (in mM) 140 KCl, 2 MgCl_2_, 1.8 CaCl_2_ and 10 HEPES (pH = 7.4).

### Single channel recording

We used an Axon200B amplifier (Axon) to measure single K^+^ channel currents of the DCT1. The currents were low-pass filtered at 1 KHz, and digitized (sampling rate of 4 KHz) by an Axon interface (Digidata 1550B). For the single channel recording, we used the pipette solution containing (in mM) 140 KCl, 2 MgCl_2_, 1 EGTA and 10 HEPES (titrated with KOH to pH = 7.4) and the bath solution containing 135 NaCl, 5 KCl, 2 MgCl_2_, 1.8 CaCl_2_, 5 glucose and 10 HEPES (titrated with NaOH to pH = 7.4). For determining the channel open probability (P_o_) and NP_o_ (a product of channel number and open probability), we selected a recording which was at least 5 min long and had less than three current levels. By doing so, we could clearly determine the channel close line thereby calculating P_o_ and NP_o ._ The following equation was used to calculate NP_o_:

$${NP}_{o}=\sum \left(t_{1}{+2t}_{2}{+......it}_{i}\right)$$

where t_i_ is the fractional open time spent at each of the observed current levels. We determined the channel conductance by measuring the current amplitudes over several voltages.

## Material

Amphotericin B was purchased from Sigma-Aldrich and TPNQ was obtained from MedChem Express. Collagenase Type 2 and L-15 medium were purchased from Worthington and Zhejiang Senrui Biotechnology Co., respectively. We obtained normal rodent diet containing 1% KCl (catalog # 2019102901), 5% KCl diet catalog #2019102901A) and 5% K-citrate diet (catalog #2019102901B) from Beijing Keao Xieli Feed Co. (Beijing, China). Both HK diets contain the same amount K^+^ (5%).

### Statistical analysis

The software (Sigma plot 14) was used for the statistical analysis. We used t-test for analyzing the values between two groups and employed paired t-test for comparisons of the values within the same group. One-way ANOVA was used for analyzing results of more than two groups and Holm-Sidak test was used as post-hoc analysis. We considered that the difference was statistically significant *if p*-values was smaller than 0.05. Data are presented as the mean ± SEM.

## Results

**FIGURE 1:**
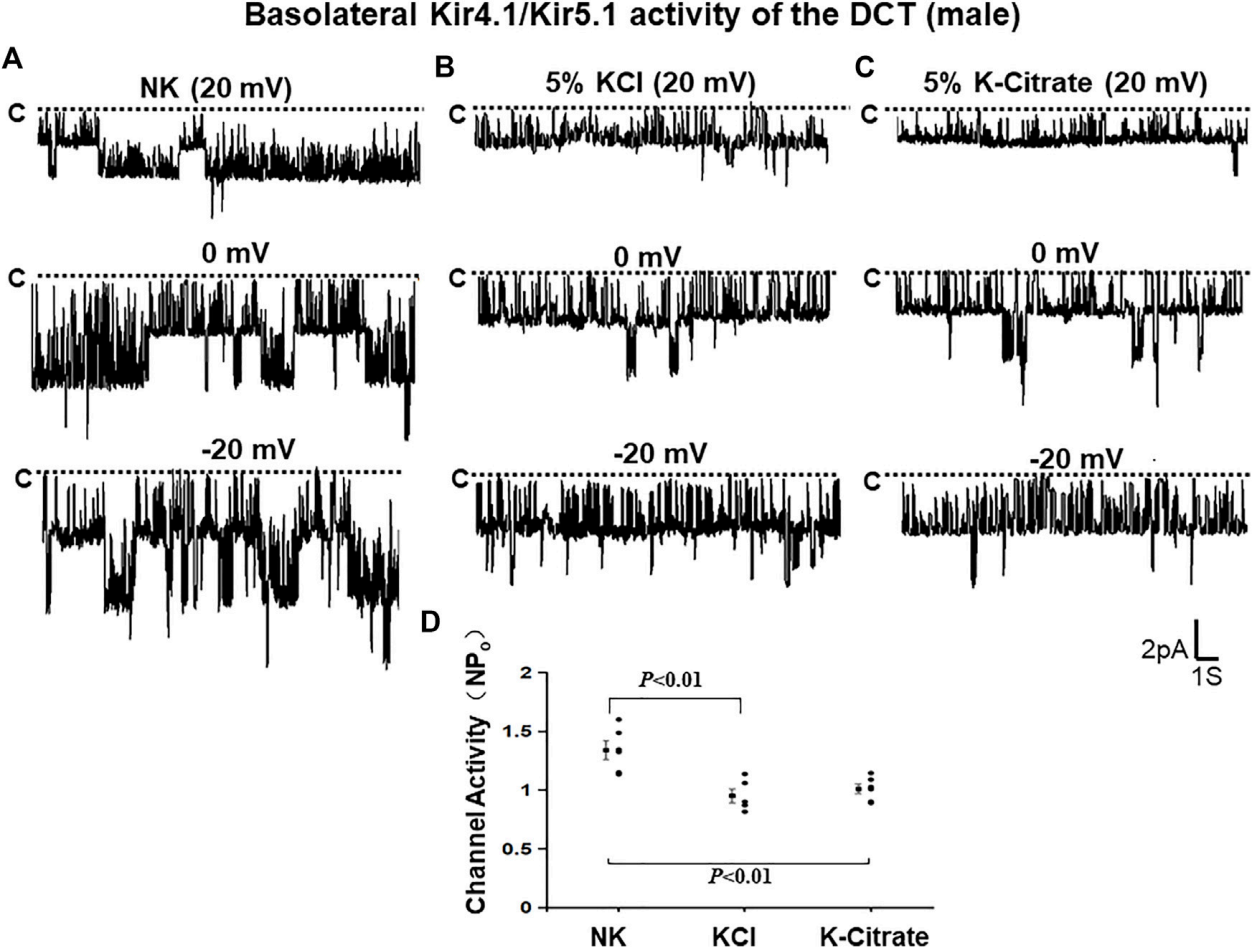
HK similarly inhibits Kir4.1/Kir5.1 of the DCT in male mice regardless Cl^−^ content. A set of single-channel recordings shows the basolateral 40 pS K^+^ channel activity in the DCT of male mice on NK (normal K^+^,1% K) **(A)**, 5% K-Cl **(B)**, and 5% K-citrate diet **(C)** for 7 days, respectively. **(D)** A scatter plot summarizing each data point and mean value of NP_o_ measured at 0 mV. The channel closed level is indicated by “C” and a dotted line. The holding potential for the patch-clamp is indicated on the top of each trace and it is 20 mV, 0 mV, and -20 mV, respectively. The bath solution contains 140 mM NaCl and 5 mM KCl and the pipette solution contains 140 mM KCl.

The effect of 5% KCl vs. K+-citrate on Kir4.1/Kir5.1 and ROMK in male mice We first used the single channel recording to examine the 40 pS K^+^ channel activity in the basolateral membrane of the DCT in the male mice on a normal K^+^ diet (NK), 5% KCl diet or 5% K^+^-citrate diet for 7 days. We have detected the 40 pS K^+^ channel in 28 patches from total 42 experiments (patches) in the male mice on NK diet (Table 1). We have calculated mean NP_o_, a product of the channel numbers (N) and open probability (P_o_) from 6 patches in which a clear channel closed level can be detected (so thus we can determine the channel numbers). Figure 1A is a representative single channel recording showing the 40 pS K^+^ channel activity at 20, 0, and -20 mV. It is apparent that 40 pS K^+^ channel activity is not voltage dependent since NP_o_ and P_o_ are similar at different holding potentials and they are 1.34 ± 0.08 and 0.37 ± 0.01, respectively. The probability of finding the 40 pS K^+^ channel was decreased in the DCT of the mice on 5% KCl for 7 days (15 patches with channel activity from total 27 patches, 56%), although the difference was not significant (Table 1). Moreover, similar results were obtained in the mice on 5% K^+^-citrate for 7 days (10 patches with channel activity from total 19 patches, 53%) (Table 1). Figures 1B,C are two sets of the single 40 pS K^+^ channel recording of the DCT in the mice on either 5% KCl and 5% K^+^-citrate, respectively. HK intake significantly decreased NP_o_ of the 40 pS K^+^ channel in the DCT (5% KCl, 0.95 ± 0.06, n = 5; 5% K^+^-citrate, 1.03 ± 0.04, n = 6) in comparison to NK. However, from the inspection of Figure 1D and Table 1, it is apparent that the difference between KCl and K^+^-citrate is statistically not significant.

**TABLE 1:** Effect of 5% KCl and K ^+^−citrate on the probability of finding Kir4.1/Kir5.1 in the basolateral membrane of the DCT, mean NPo and mean Po per patch in the male mice. Asterisk indicates the significant difference in comparison to the group on NK. Numbers.

	Number of total patches	Numbers of patches with Kir4.1/Kir5.1	Mean NPo of Kir4.1/Kir5.1 Per patch	Mean Po of Kir4.1/Kir5.1 Per patch
Normal K^+^ (NK)	42	28 (67%)	1.34 ± 0.08 (n = 6)	0.37 ± 0.01 (n = 6)
5% KCl	27	15 (56%)	0.95 ± 0.06 * (n = 5)	0.32 ± 0.02 (n = 5)
5% K + - citrate	19	10 (53%)	1.03 ± 0.04 * (n = 6)	0.31 ± 0.02 (n = 6)

**FIGURE 2:**
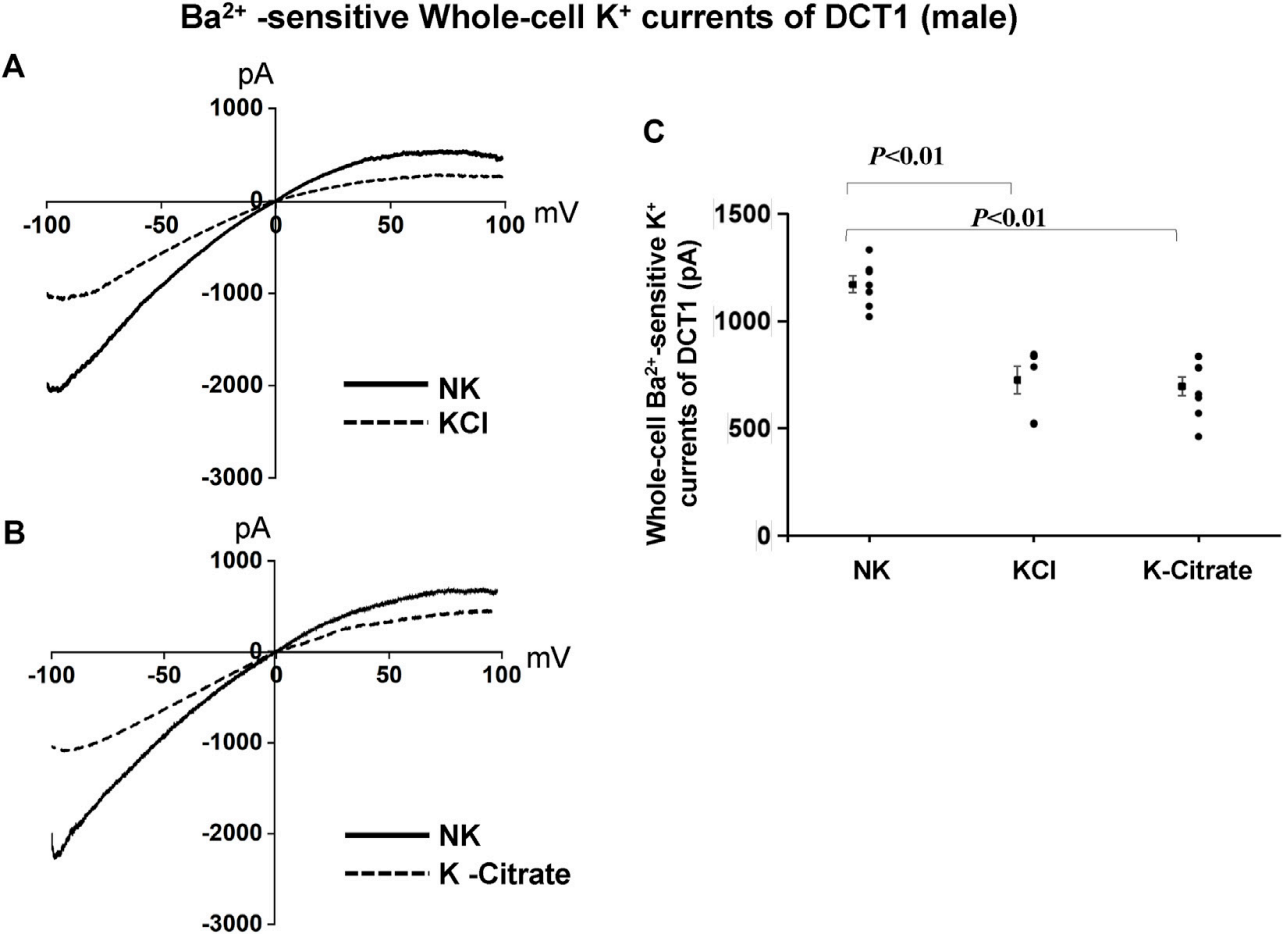
Cl^−^ content of HK diets does not affect HK-induced inhibition of Kir4.1/Kir5.1 currents of the DCT of male mice. A set of whole-cell recordings shows Ba^2+^(0.1 mM)-sensitive Kir4.1/Kir5.1-mediated K^+^ currents in the DCT of male mice on NK or 5% K-Cl diet for 7 days **(A)** and on NK and K-citrate for 7 days **(B)**. The whole-cell K^+^ currents were measured with ramp protocol from -100 to 100 mV using symmetrical 140 mM KCl solution in the bath and pipette. **(C)** A scatter graph summarizes the results of K^+^ currents measured at –60 mV in the DCT of male mice on different K^+^ diets. The mean value and SEM are shown on the left of each column.

**FIGURE 3:**
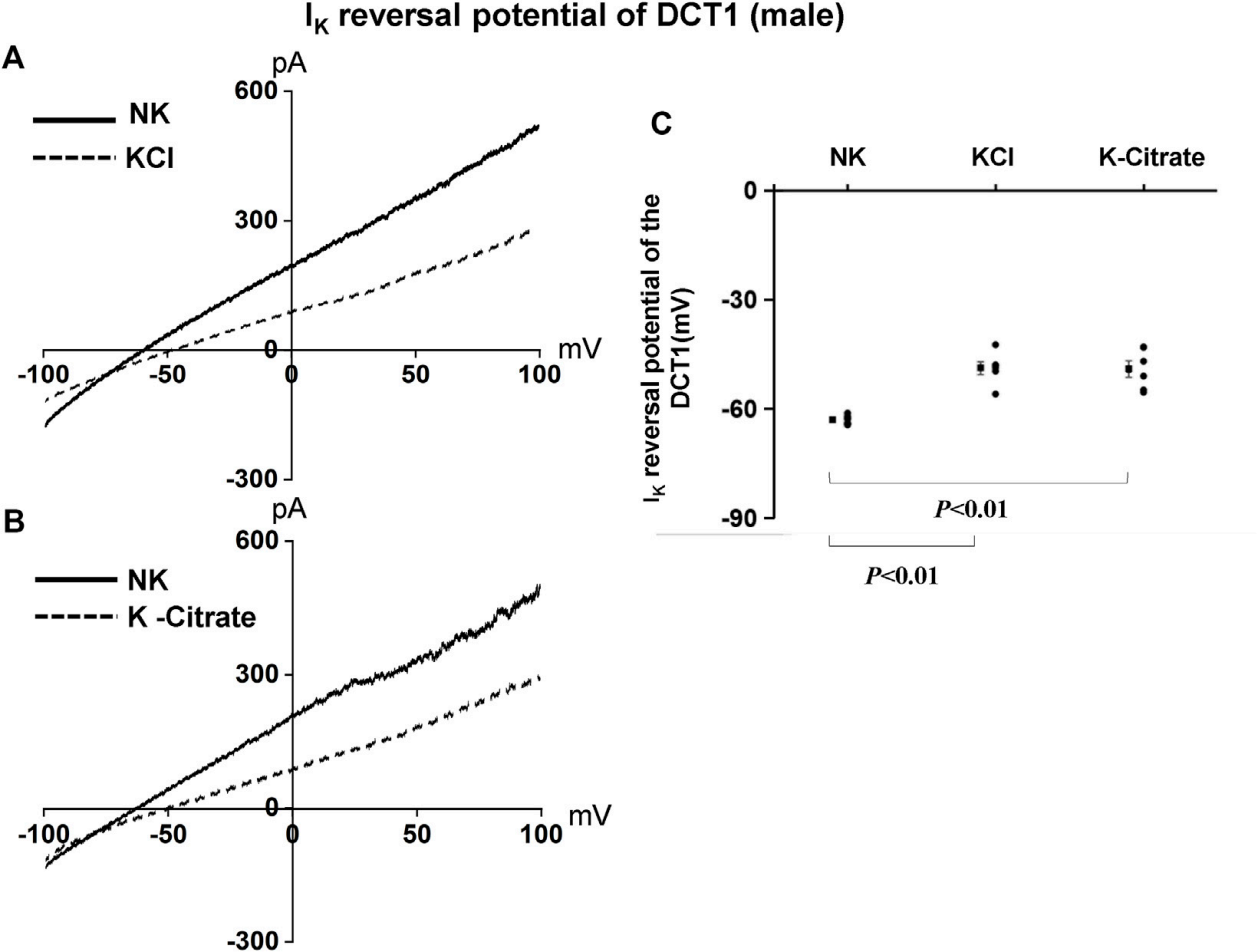
HK similarly depolarizes the basolateral membrane of the DCT in male mice regardless Cl^−^ content. A set of whole-cell recordings shows I_K_ reversal potential in the DCT of male mice on NK or 5% K-Cl diet for 7 days **(A)** and on NK or 5% K-citrate diet for 7 days **(B)**. For the measurement of I_K_ reversal potential, the bath solution contained 140 mM NaCl and 5 mM KCl, and the pipette solution contained 140 mM KCl. **(C)** A scatter graph summarizes the results of experiments in which I_K_ reversal potentials were measured in the DCT of male mice on different K^+^ diets. The mean value and SEM are shown on the left of each column.

We have also used the whole-cell recording to examine Kir4.1/Kir5.1-mediated K^+^ currents in the DCT1. Because Kir4.1/Kir5.1 is the only type K^+^ channels in the DCT1 (Wu et al., 2020a), the Ba^2+−^sensitive whole-cell K^+^ currents of DCT1 are a reliable index for Kir4.1/Kir5.1 activity. Figures 2A,B are two representative whole-cell recordings showing Kir4.1/Kir5.1-mediated K^+^ currents measured with ramp protocol from -100 to 100 mV in the DCT1 of the mice on 5% KCl and 5% K^+^-citrate, respectively. Figure 2C is a scatter plot summarizing each data point measured at -60 mV and the corresponding mean value is shown at left side of each column. The whole-cell K^+^ currents were 1,170 ± 40 pA at -60 mV under NK conditions (*n* = 7) whereas two HK diets for 7 days decreased the Ba^2+^-sensitive Kir4.1/Kir5.1-mediated K^+^ currents equally (5% KCl, 725 ± 65 pA at -60 mV, *n* = 6; 5% K^+^-citrate, 700 ± 43 pA at -60 mV, *n* = 8). Since Kir4.1/Kir5.1 determines the membrane potential of DCT1, a decrease in Kir4.1/Kir5.1 activity is expected to decrease the negativity of the DCT1 membrane potential (depolarization). Thus, we next used the whole-cell recording to measure K^+^ currents (I_K_)-reversal potential of the DCT1, an index of the membrane potential. Figures 3A,B are two sets of the recording showing the I_K_ reversal potential of the DCT1 of the mice on 5% KCl and 5% K^+^-citrate, respectively. Figure 3C is a scatter plot summarizing each data point and the corresponding mean value at left side of each column. The I_K_-reversal potential of the DCT1 in the mice on NK was -63 ± 0.5 mV (*n* = 7) and it was -49 ± 1.8 mV (*n* = 6) in the mice on 5% KCl for 7 days and -49 ± 2.3 mV (*n* = 6) in the mice on 5% K^+^-citrate. Thus, HK diets with either 5% KCl or 5% K-citrates similarly inhibited the Kir4.1/Kir5.1 activity and depolarized the DCT membrane potential.

**FIGURE 4:**
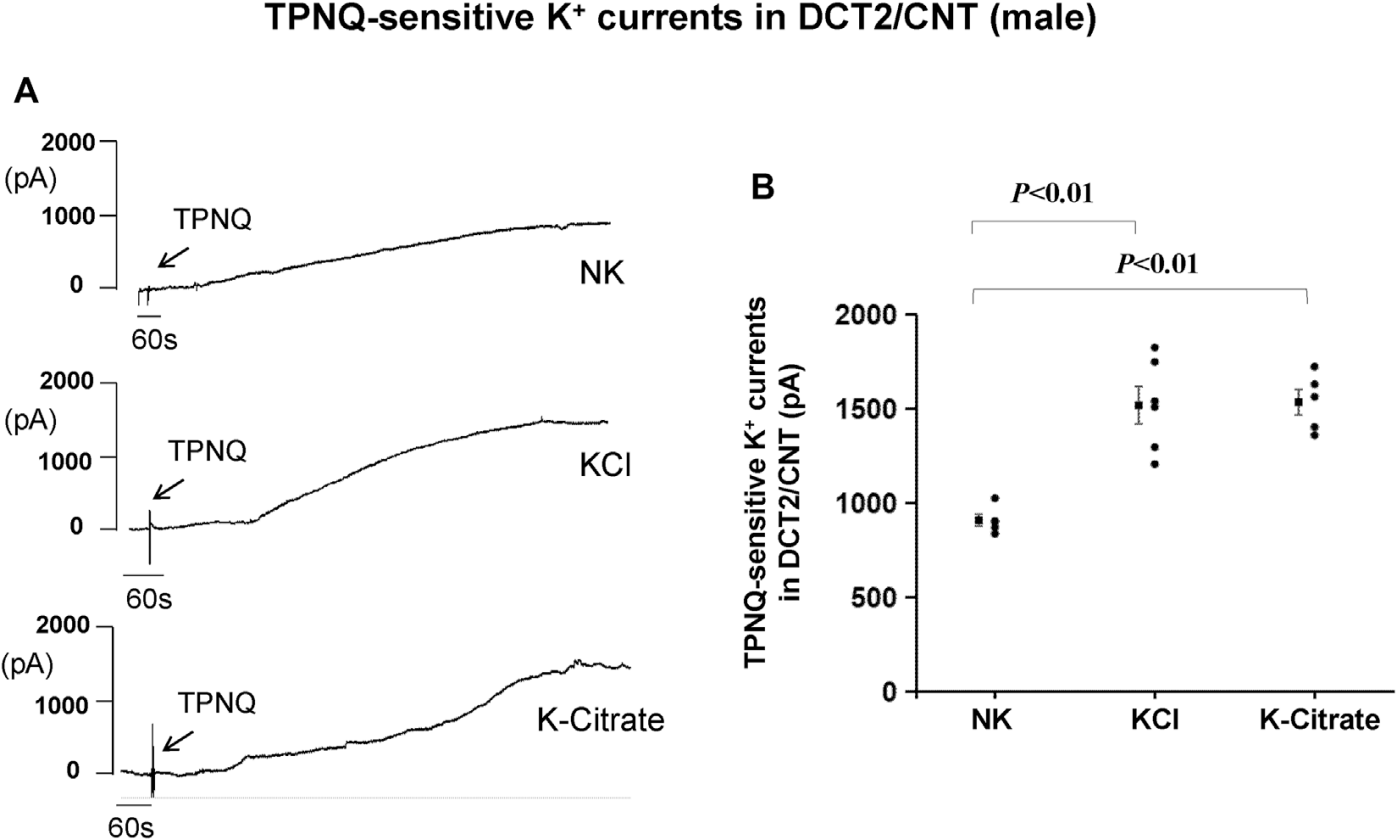
HK intake stimulates ROMK in the DCT2/CNT of male mice regardless Cl^−^ content. **(A)** A set of whole-cell recordings shows TPNQ (400 nM)-sensitive (ROMK-mediated) K^+^ currents in the DCT2/CNT of the male mice on NK, 5% K-Cl, and 5% K-citrate diet for 7 days, respectively. The K^+^ currents were measured with gap-free protocol at −40 mV using symmetrical 140 mM KCl solution in the bath and pipette. **(B)** A scatter graph summarizes the values measured at –40 mV in the DCT2/CNT of male mice on different K^+^ diets. The mean value and SEM are shown on the left of each column.

While Kir4.1/Kir5.1 heterotetramer is expressed in the basolateral membrane of the DCT, the ROMK channel is expressed in the apical membrane of the DCT2 (Yang et al., 2021; Zhang et al., 2021; Nesterov et al., 2022). Previous studies have shown that HK intake stimulates ROMK channel activity in the DCT2 (Wu et al., 2020b; Yang et al., 2021). To assess whether the Cl^−^ content in HK diet affects the effect of HK diet on ROMK in the DCT2, we used the whole-cell recording to measure TPNQ-sensitive K^+^ currents (ROMK) in the split-open DCT2 and initial connecting tubule (CNT). Figure 4A is a set of recordings showing whole-cell TPNQ-sensitive K^+^ currents (ROMK) measured at -40 mV using gap-free protocol and Figure 4B is a scatter plot summarizing each data point and the mean value at the left side of each column. HK diet for 7 days significantly increased ROMK activity in the DCT2/CNT from the control value (910 ± 32 pA, *n* = 5) to 1,520 ± 99 pA (5% KCl, *n* = 6) and 1,540 ± 68 pA (5% K^+^-citrate, *n* = 5). Thus, HK-induced stimulation of ROMK in the DCT2/initial CNT is similar in the male mice on 5% KCl or 5% K^+^-citrate.

### The effect of 5% KCl vs. K^+^-citrate on Kir4.1/Kir5.1 and ROMK in female mice

**FIGURE 5:**
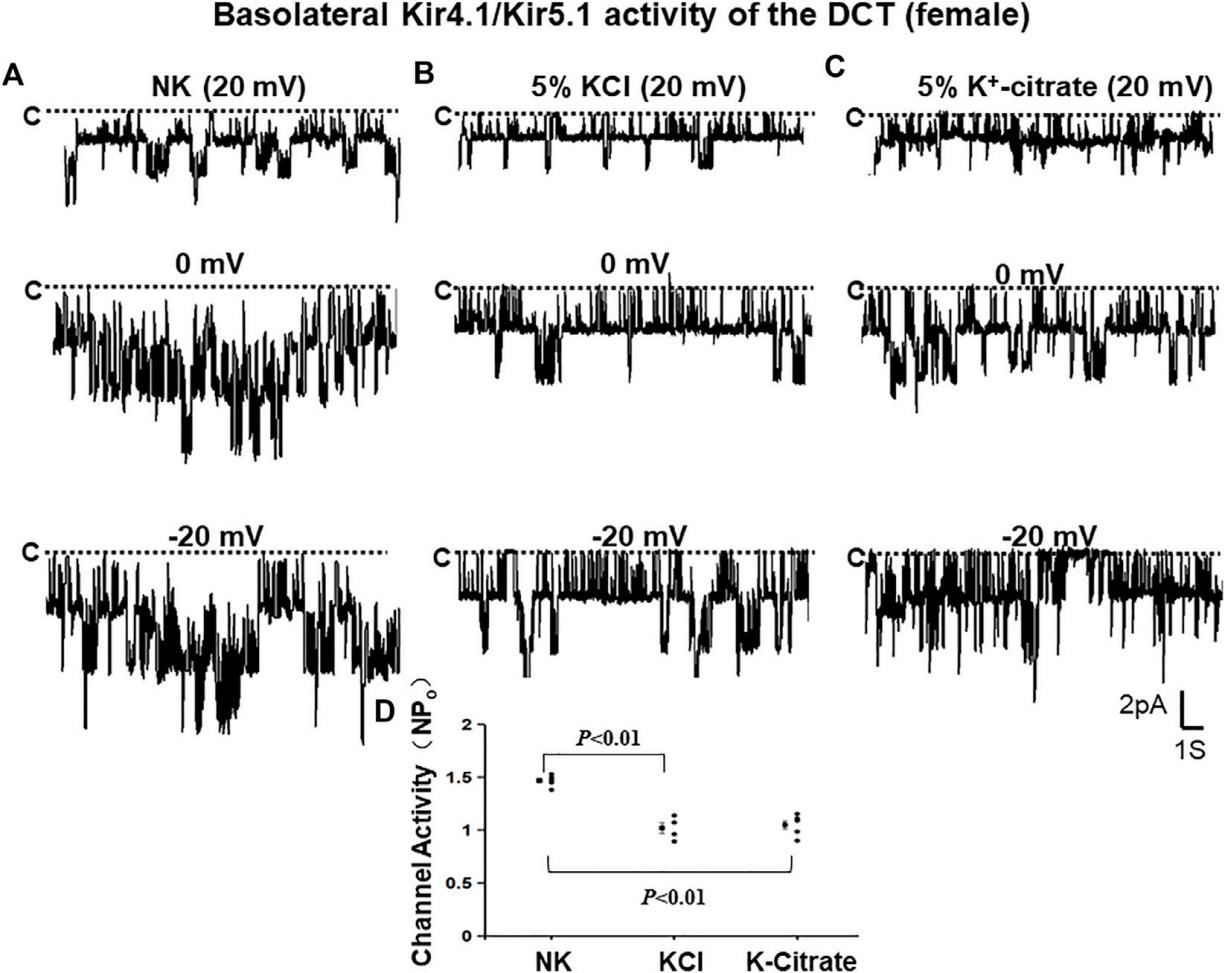
HK similarly inhibits Kir4.1/Kir5.1 of the DCT in female mice regardless Cl^−^ content. A set of single-channel recordings shows the basolateral 40 pS K^+^ channel activity in the DCT of female mice on NK (normal K^+^,1% K) **(A)**, 5% K-Cl **(B)**, and 5% K-citrate diet **(C)** for 7 days, respectively. **(D)** A scatter plot summarizing each data point and mean value of NP_o_ measured at 0 mV. The channel closed level is indicated by “C” and a dotted line. The holding potential for the patch-clamp is indicated on the top of each trace and it is 20 mV, 0 mV, and -20 mV, respectively. The bath solution contains 140 mM NaCl and 5 mM KCl and the pipette solution contains 140 mM KCl.

To examine whether Cl^−^ content of HK diets plays a role in mediating the effect of increased K^+^ intake on Kir4.1/Kir5.1 in the DCT of female mice, we first used the single channel recording to examine the 40 pS K^+^ channel activity in the basolateral membrane of the DCT of the female mice on NK, 5% KCl diet or 5% K^+^-citrate diet for 7 days. We have detected the 40 pS K^+^ channel in 8 patches from total 12 experiments (patches) in the female mice on NK diet (Table 2). Figure 5A is a representative single channel recording showing the 40 pS K^+^ channel activity of the DCT at 20, 0, and -20 mV in the female animals on NK. We have calculated mean NP_o_ from 5 patches in which a clear channel closed level can be detected. Again, the 40 pS K^+^ channel activity of the DCT in female mice was also not voltage dependent since NP_o_ and P_o_ are similar at different holding potentials and they are 1.47 ± 0.02 and 0.37 ± 0.01, respectively. Moreover, the probability of finding the 40 pS K^+^ channel tends to be decreased in the DCT of the mice on 5% KCl for 7 days in comparison to NK (13 patches with channel activity from total 23 patches, 57%) but the difference was not significant (Table 2). Moreover, similar results were obtained in the mice on 5% K^+^-citrate for 7 days (24 patches with channel activity from total 41 patches, 59%) (Table 2). Figures 5B,C are two sets of the single 40 pS K^+^ channel recording of the DCT in the mice on either 5% KCl and 5% K^+^-citrate, respectively. HK intake significantly decreased NP_o_ of the 40 pS K^+^ channel in the DCT of the female mice (5% KCl, 1.02 ± 0.05, *n* = 4; 5% K^+^-citrate, 1.05 ± 0.04, *n* = 5) in comparison to NK. However, from the inspection of Figure 5D and Table 2, it is apparent that the difference between KCl and K^+^-citrate is statistically not significant.

**TABLE 2:** Effect of 5% KCl and K + -citrate on the probability of finding Kir4.1/Kir5.1 in the basolateral membrane of the DCT, mean NPo and mean Po per patch in the female mice. Asterisk indicates the significant difference in comparison to the group on NK.

	Number of total patches	Numbers of patches with Kir4.1/Kir5.1	Mean NPo of Kir4.1/Kir5.1 Per patch	Mean Po of Kir4.1/Kir5.1 Per patch
Normal K^+^ (NK)	12	8 (67%)	1.47 ± 0.02 (n = 5)	0.37 ± 0.01 (n = 5)
5% KCl	23	13 (57%)	1.02 ± 0.05 * (n = 4)	0.34 ± 0.01 (n = 4)
5% K^+^ - citrate	41	24 (59%)	1.05 ± 0.04 * (n = 5)	0.35 ± 0.02 (n = 5)

**FIGURE 6:**
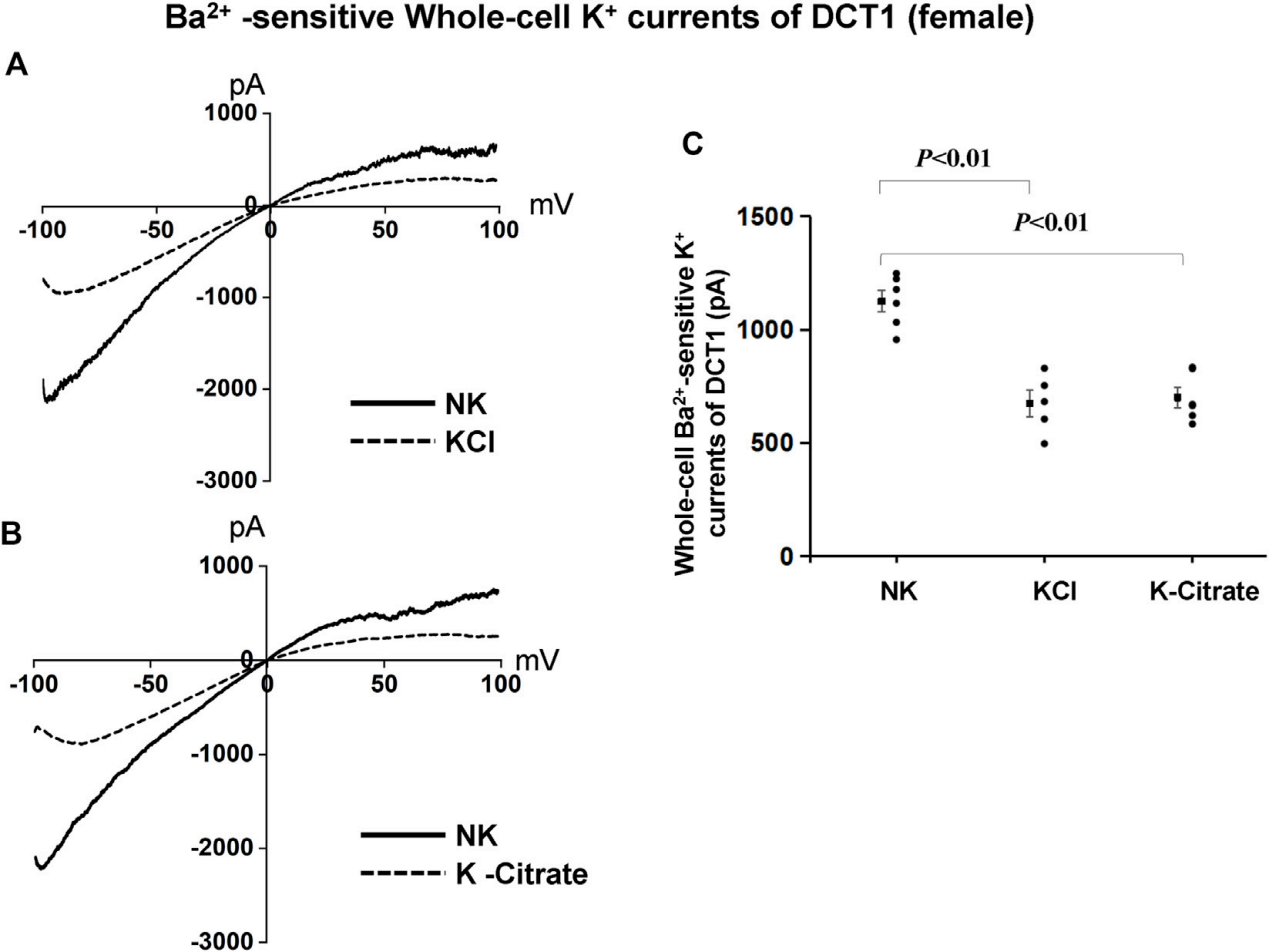
Cl− content of HK diets does not affect HK-induced inhibition of Kir4.1/Kir5.1 currents of the DCT of female mice. A set of whole-cell recordings shows Ba^2+^(0.1 mM)-sensitive Kir4.1/Kir5.1-mediated K^+^ currents in the DCT of female mice on NK or 5% K-Cl diet for 7 days **(A)** and on NK and K-citrate for 7 days **(B).** The whole-cell K^+^ currents were measured with ramp protocol from −100 to 100 mV using symmetrical 140 mM KCl solution in the bath and pipette. **(C)** A scatter graph summarizes the results of K^+^ currents measured at –60 mV in the DCT of female mice on different K^+^ diets. The mean value and SEM are shown on the left of each column.

**FIGURE 7:**
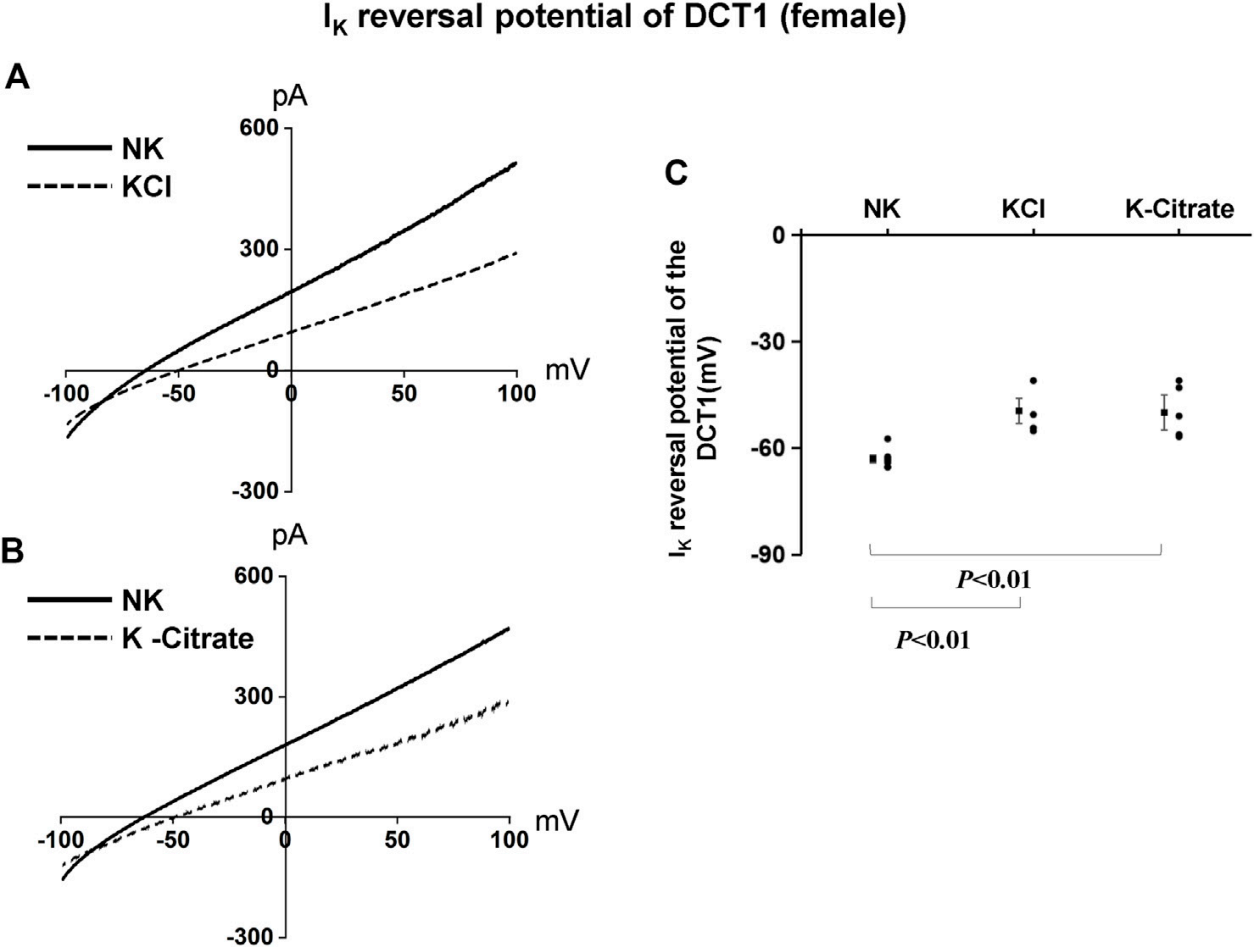
HK similarly depolarizes the basolateral membrane of the DCT in female mice regardless Cl− content. A set of whole-cell recordings shows I_K_ reversal potential in the DCT of female mice on NK or 5% K-Cl diet for 7 days **(A)** and on NK or 5% K-citrate diet for 7 days **(B)**. For the measurement of I_K_ reversal potential, the bath solution contained 140 mM NaCl and 5 mM KCl, and the pipette solution contained 140 mM KCl. **(C)** A scatter graph summarizes the results of experiments in which I_K_ reversal potentials were measured in the DCT of female mice on different K^+^ diets. The mean value and SEM are shown on the left of each column.

We have next used the whole-cell recording to examine Kir4.1/Kir5.1-mediated K^+^ currents in the DCT1 of female mice on NK and HK diets for 7 days. Figures 6A,B are two representative whole-cell recordings showing Kir4.1/Kir5.1-mediated K^+^ currents measured with ramp protocol from -100 to 100 mV in the DCT1 of the female mice on 5% KCl and 5% K^+^-citrate, respectively. Figure 6C is a scatter plot summarizing each data point measured at -60 mV and the corresponding mean value is shown at left side of each column. The whole-cell K^+^ currents were 1,125 ± 47 pA at -60 mV under control conditions (NK) (*n* = 6) whereas two HK diets for 7 days decreased the Ba^2+^-sensitive Kir4.1/Kir5.1-mediated K^+^ currents equally (5% KCl, 674 ± 58 pA at -60 mV, *n* = 5; 5% K^+^-citrate, 700 ± 43 pA at -60 mV, *n* = 6). We next used the whole-cell recording to measure I_K_-reversal potential of the DCT1 of the female mice on NK and HK diet for 7 days. Figures 7A,B are two sets of the recording showing the I_K_ reversal potential of the DCT of the mice on NK and 5% KCl or 5% K^+^-citrate, respectively. Figure 7C is a scatter plot summarizing each data point and the corresponding mean value at left side of each column. The I_K_-reversal potential of the DCT1 in the female mice on NK was -63 ± 1 mV (*n* = 7) and it was -50 ± 4 mV (*n* = 4) in the mice on 5% KCl for 7 days and -50 ± 3 mV (*n* = 5) in the mice on 5% K^+^-citrate. Thus, similar to male mice, HK diets with either 5% KCl or 5% K^+^-citrates similarly inhibited the Kir4.1/Kir5.1 activity and depolarized the DCT membrane potential in the female mice.

**FIGURE 8:**
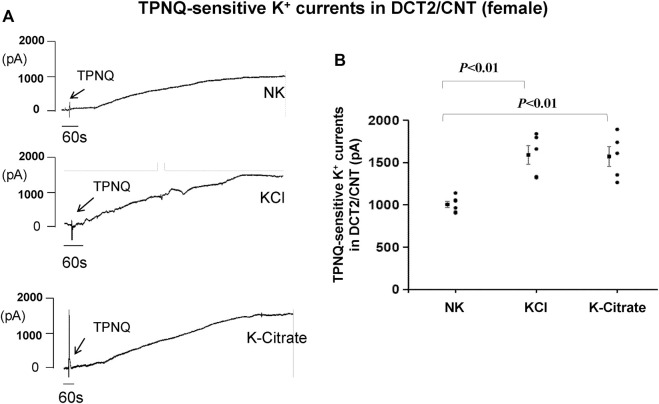
HK intake stimulates ROMK in the DCT2/CNT of female mice regardless Cl− content. **(A)** A set of whole-cell recordings shows TPNQ (400 nM)-sensitive (ROMK-mediated) K^+^ currents in the DCT2/CNT of the female mice on NK, 5% K-Cl, and 5% K-citrate diet for 7 days, respectively. The K^+^ currents were measured with gap-free protocol at −40 mV using symmetrical 140 mM KCl solution in the bath and pipette. **(B)** A scatter graph summarizes the values measured at –40 mV in the DCT2/CNT of female mice on different K^+^ diets. The mean value and SEM are shown on the left of each column.

To test whether the Cl^−^ content in HK diet affects the effect of HK diet on ROMK in the DCT2/initial CNT of the female mice, we used the whole-cell recording to measure TPNQ-sensitive K^+^ currents (ROMK) in the split-open DCT2 and initial CNT. Figure 8A is a set of recordings showing whole-cell TPNQ-sensitive K^+^ currents (ROMK) measured at -40 mV using gap-free protocol and Figure 8B is a scatter plot summarizing each data point and the mean value at the left side of each column. HK diet for 7 days significantly increased ROMK activity in the DCT2/CNT from the control value (1,005 ± 40 pA, *n* = 6) to 1,590 ± 112 pA (5% KCl, *n* = 5) and 1,570 ± 117 pA (5% K^+^-citrate, *n* = 5). Thus, HK-induced stimulation of ROMK in the DCT2/initial CNT is similar in the female mice on 5% KCl or 5% K^+^-citrate.

### The effect of HK on Kir4.1/Kir5.1 and ROMK is similar in male and female mice

**FIGURE 9:**
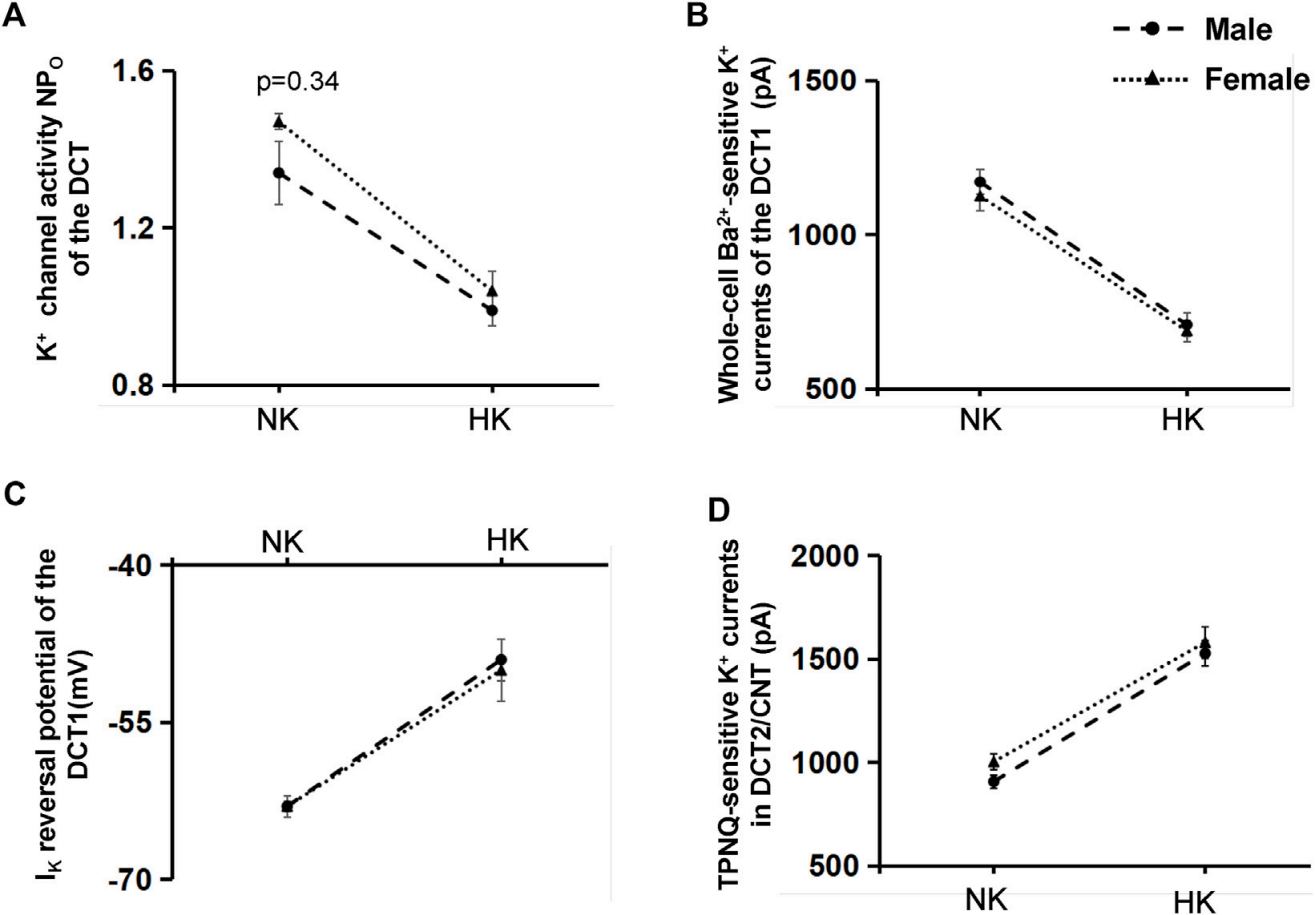
The effect of HK on Kir4.1/Kir5.1 and ROMK of DCT is not affected by gender. **(A)** A Line graphs summarizes the experiments in which NP_o_ of the 40 pS K^+^ channel (Kir4.1/Kir5.1) was measured in the DCT of male (circle) and female (triangle) mice on NK or HK (5% KCl and 5% K-citrate) for 7 days. **(B)** A line graphs summarizes the experiments in which whole-cell Kir4.1/Kir5.1-mediated K^+^ currents at −60 mV were measured in the DCT of male (circle) and female (triangle) mice on NK or HK (5% KCl and 5% K-citrate) for 7 days. **(C)** A line graphs summarizes the experiments in which I_K_ reversal potential was measured in the DCT of male (circle) and female (triangle) mice on NK or HK (5% KCl and 5% K-citrate) for 7 days. **(D)** A line graphs summarizes the experiments in which whole-cell TPNQ-sensitive K^+^ currents (ROMK) at −40 mV were measured in the DCT2/CNT of male (circle) and female (triangle) mice on NK or HK (5% KCl and 5% K-citrate) for 7 days.

After demonstrating that Cl^−^ content of HK diet does not affect HK-induced inhibition of Kir4.1/Kir5.1 of the DCT and HK-induced stimulation of ROMK in the DCT2/initial CNT, we examined whether gender has an effect on HK-induced inhibition of Kir4.1/Kir5.1 and HK-induced stimulation of ROMK. Figure 9A is a line graph summarizing the 40 pS K^+^ channel activity in the male and female mice on NK or HK (including 5% KCl and 5% K^+^-citrate). The NP_o_ in the male mice on NK is 1.34 ± 0.08 (*n* = 6), which is not significantly different from female mice on NK (1.47 ± 0.02, *n* = 5). The NP_o_ in the male mice on HK is 0.99 ± 0.04 (*n* = 11), which is also not significantly different from female mice on HK (1.04 ± 0.05, *n* = 9). Figure 9B is a line graph summarizing the results in which the Ba^2+^-sensitive whole-cell K^+^ currents at -60 mV were measured in the DCT1 of male and female mice on NK and HK (including 5% KCl and 5% K^+^-citrate). The Kir4.1/Kir5.1-mediated K^+^ currents of male mice on NK were 1,170 ± 40 pA (*n* = 7), which is similar to those in female mice on NK (1,125 ± 47 pA, *n* = 6). The Kir4.1/Kir5.1-mediated K^+^ currents of male mice on HK were 710 ± 40 pA (*n* = 14), which is similar to those in female mice on HK (680 ± 34 pA, *n* = 11). Figure 9C is a light graph summarizing I_K_-reversal potential measured in the DCT1 of male and female mice on NK and HK (including 5% KCl and 5% K^+^-citrate). I_K_-reversal potential of the DCT1 in male mice on NK is -63 ± 0.5 mV (*n* = 7) whereas it is also -63 ± 1 mV (*n* = 7) in female mice on NK. I_K_-reversal potential of the DCT1 in male mice on HK is -49 ± 1 mV (*n* = 12) whereas it is -50 ± 2 mV (*n* = 9) in female mice on HK. Thus, the effect of HK on NP_o_, Kir4.1/Kir5.1-mediated K^+^ current and DCT1 membrane potential is similar in male and female mice. Finally, gender also does not affect the HK-induced stimulation of ROMK channels in the DCT2/CNT. Figure 9D is a line graph summarizing the results in which the TPNQ-sensitive whole-cell K^+^ currents (ROMK) at -40 mV were measured in the DCT2/CNT of male and female mice on NK and HK (including 5% KCl and 5% K^+^-citrate). The ROMK-mediated K^+^ currents of male mice on NK were 910 ± 32 pA (*n* = 5), which is similar to those in female mice on NK (1,005 ± 40 pA, *n* = 6). The ROMK-mediated K^+^ currents of male mice on HK were 1,530 ± 59 pA (*n* = 11), which is similar to those in female mice on HK (1,580 ± 76 pA, *n* = 10).

## Discussion

Previous studies have convincingly demonstrated that Kir4.1 and Kir5.1 are expressed in the DCT (Tucker et al., 2000; Derst et al., 2001; Lourdel et al., 2002; Huang et al., 2007; Cuevas et al., 2017; Palygin et al., 2017). Moreover, Kir4.1 interacts with Kir5.1 in the basolateral membrane to form a heterotetramer, a 40 pS inwardly-rectifying K^+^ channel in the DCT (Lachheb et al., 2008; Zhang et al., 2013; Zhang et al., 2014). The role of Kir4.1 and Kir5.1 in forming the only type basolateral K^+^ channels in the DCT is different: Kir4.1 is responsible for providing the K^+^ conductance (Cuevas et al., 2017; Wang et al., 2018a), whereas Kir5.1 serves as a regulatory subunit for the heterotetramer (Tanemoto et al., 2000; Tucker et al., 2000; Pessia et al., 2001; Casamassima et al., 2003; Wang et al., 2018b). Furthermore, our study have demonstrated that Kir5.1 interacts with the E3 ubiquitin ligase Nedd4-2, which then regulates Kir4.1 ubiquitination (Wang et al., 2018b). Because Kir4.1/Kir5.1 heterotetramer determines the cell membrane potential of the DCT (Terker et al., 2015; Su and Wang, 2016; Su et al., 2019), changes in Kir4.1/Kir5.1 activity should affect the driving force for eletrogenic Cl^−^ movement through basolateral Cl^−^ channels (Terker et al., 2015). Thus, the stimulation of Kir4.1/Kir5.1 activity in the DCT promoting intracellular Cl^−^ exit across the basolateral membrane thereby decreasing the intracellular Cl^−^ concentration and stimulating Cl^−^-sensitive WNK (with-no-lysine kinase) activity. High WNK activity is expected to increase thiazide-sensitive NCC activity. On the other hand, decreased Kir4.1/Kir5.1 activity in the DCT is expected to raise intracellular Cl^−^ thereby decreasing WNK activity and inhibiting NCC. Indeed, Su et al. (2020) have recently demonstrated that the inhibition of Kir4.1/Kir5.1 channels increased the intracellular Cl^−^ concentrations of the DCT. The view that basolateral Kir4.1/Kir5.1 activity of the DCT controls NCC activity/expression by regulating [Cl_i_^−^] is also supported by the observations that an increase in the intracellular Cl^−^ concentration due to depolarization of the membrane voltage are responsible for the suppression of NCC activity in DCT cells which were transfected with loss-function-of-Kir4.1 mutants (Terker et al., 2015).

Previous studies have convincingly demonstrated that the NCC activity is regulated by dietary K^+^ intake such that NCC activity is stimulated in the animals on decreased dietary K^+^ intake whereas NCC activity is inhibited by increased dietary K^+^ intake (van der Lubbe et al., 2013; Castaneda-Bueno et al., 2014; Rengarajan et al., 2014; Terker et al., 2015; Terker et al., 2016; Wang et al., 2018a; Yang et al., 2018). The low dietary K^+^ intake-induced stimulation of NCC activity is essential for preserving K^+^ during hypokalemia because high NCC activity should decrease Na^+^ and volume delivery to the ASDN thereby inhibiting ENaC-dependent K^+^ excretion. Whereas high-dietary K^+^ intake-induced inhibition of NCC is important to facilitate ENaC-dependent renal K^+^ excretion during hyperkalemia (Ellison et al., 2015), because low NCC activity in the DCT should increase the Na^+^ delivery to the ASDN thereby enhancing ENaC-dependent renal K^+^ excretion. Terker et al. (2016) have used phosphorylated NCC as an index of NCC activation, they have observed that there is a steep relationship between plasma K^+^ concentration and NCC activity between 3.5 and 4.5 mM plasma K^+^. This suggests the importance of NCC activity in maintaining K^+^ homeostasis and renal K^+^ excretion because plasma K^+^ concentrations between 3.5 and 4.5 mM falls exactly in physiological relevant ranges.

By performing electrophysiological studies in the mouse DCT, our previous study has demonstrated that high K^+^ intake depolarizes the membrane of the DCT whereas low K^+^ intake hyperpolarizes membrane of the DCT (Wang et al., 2018a). Furthermore, both Kir4.1 and Kir5.1 in the DCT are required for dietary K^+^ intake-induced changes in the basolateral K^+^ permeability and DCT membrane potential since (Wang et al., 2018a; Wu et al., 2019). Although increased dietary K^+^ intake-induced inhibition of Kir4.1/Kir5.1 and DCT membrane potential is well established (Wang et al., 2018a; Wu et al., 2019), the role of Cl^−^ content of HK diet in decreasing the negativity of the DCT membrane potential is not tested. This is due to the fact that the HK diets used in the previous experiments often also contains high Cl^−^ contents (Wang et al., 2018a; Wu et al., 2019). It is not known whether increased Cl^−^ content in the HK diet plays a role in causing depolarization. Three observations of our present study have suggested that high Cl^−^ content in HK diet did not affect the HK-induced depolarization of DCT membrane potential. First, both 5% KCl and K^+^ -citrate equally decreased NP_o_ of the 40 pS K^+^ channel to a similar extend. Secondly, both high K^+^ diets similarly decreased the whole-cell Kir4.1/Kir5.1-mediated K^+^ currents of the DCT to the identical levels. Finally, HK intake similarly depolarized the DCT membrane potential regardless the Cl^−^ content. Thus, Cl^−^ content of the HK diet has no effect on HK-induced inhibition of Kir4.1/Kir5.1 in the DCT.

We have also demonstrated that the baseline Kir4.1/Kir5.1 activity and HK-intake-induced inhibition of Kir4.1/Kir5.1 activity in the DCT are similar in the male and female mice. It is well demonstrated that female mice have a higher NCC activity /expression than in male mice (Veiras et al., 2017). Since Kir4.1/Kir5.1 in the DCT plays an important role in regulating NCC activity/expression (Su et al., 2019), it raises the possibility whether female mice have also a higher baseline-Kir4.1/Kir5.1 activity and a higher negativity of the DCT membrane potential than in male mice. However, our present study has excluded this possibility that higher NCC activity/expression in female mice than in male mice is the result of more activated Kir4.1/Kir5.1 channels in female animals than in male animals. First, the probability of finding the 40 pS K^+^ channel is the same between male and female mice under the control conditions. Secondly, the baseline whole-cell Kir4.1/Kir5.1-mediated K^+^ currents are similar between male and female mice. Finally, the DCT membrane potential has the same the negativity in both male and female mice. Thus, the present study excludes the possibility that higher NCC activity in female mice than in male mice is due to higher DCT membrane potential in female mice than male mice.

The third finding of the present study is to demonstrate that HK-induced stimulation of ROMK channel activity in DCT2/CNT is not affected by Cl^−^ content of HK diets and that baseline ROMK channel activity and HK-induced stimulation of ROMK are also similar between male and female mice. ROMK channel in the ASDN plays an important role in mediating renal ENaC-dependent K^+^ excretion (Wang et al., 1997; Giebisch, 1999). Moreover, two recent studies combining *in vivo* experiments and mathematic modeling have convincingly demonstrated that DCT2 and CNT are two main nephron segments responsible not only for controlling renal K^+^ excretion under control conditions but also the main segment for facilitating renal K^+^ excretion during HK (Yang et al., 2021; Nesterov et al., 2022). This notion is based on the finding that both ROMK and ENaC activity are significantly higher in the DCT2 and CNT than in the CCD. Thus, the observation that baseline ROMK channel activity and HK-induced stimulation of ROMK in the DCT2/CNT are similar between male and female mice would exclude the possibility that the different ROMK activity in the DCT2 and CNT may contribute to a possible gender difference for renal K^+^ excretion. We conclude that Cl^−^ content of the HK diets has no effect on HK-induced inhibition of Kir4.1/Kir5.1 of the DCT and HK-induced stimulation of ROMK in the DCT2/CNT. Also, baseline Kir4.1/Kir5.1 activity in the DCT and ROMK in the DCT2/CNT and the effect of HK on Kir4.1/Kir5.1 and ROMK are the same between male and female mice.

## Data Availability

The original contributions presented in the study are included in the article/Supplementary material, further inquiries can be directed to the corresponding authors.
